# Rituals, ceremonies and customs related to sacred trees with a special reference to the Middle East

**DOI:** 10.1186/1746-4269-3-28

**Published:** 2007-07-09

**Authors:** Amots Dafni

**Affiliations:** 1 Institute of Evolution, Haifa University, Haifa 31905, Israel

## Abstract

Tree worship is very common worldwide. This field study surveys the ceremonies and customs related to sacred trees in present-day Israel; it includes the results of interviews with 98 informants in thirty-one Arab, Bedouin, and Druze villages in the Galilee.

The main results are:

1. Sacred trees were treated as another kind of sacred entity with all their metaphysical as well as physical manifestations.

2. There is not even one ceremony or custom that is peculiar only to a sacred tree and is not performed in other sacred places (such as a saint's grave or a mosque).

3. Few customs, such as: quarrel settling (= Sulkha), leaving objects to absorb the divine blessing and leaving objects for charity) seem to be characteristic of this region, only.

4. In modern times, sacred trees were never recorded, in Israel, as centres for official religious ceremonies including sacrifices, nor as places for the performing of rites of passage.

5. There is some variation among the different ethnic groups: Kissing trees and worshipping them is more common among the Druze although carrying out burials under the tree, leaving water and rain-making ceremonies under them have not been recorded in this group. Passing judgments under the tree is more typical of the Bedouin in which the sacred trees were commonly used as a public social centre.

Most of the customs surveyed here are known from other parts of the world. The differences between Muslims and Druze are related to the latter's belief in the transmigration of souls.

## Background

On the subject of sacred places, Turner [[1]:24] states: "This place where other realms are meet is also indicated by various forms representing a link or connection between the human and transhuman spheres, and usually set in a vertical dimension as a ladder, poles and pillars, trees and hills". Sacred places are found all over the globe and may consist of various artificial objects (buildings, shrines, graves) as well as natural ones (mountains, water source and trees).

According to Eliade [[2]:26] "Every sacred space implies a hierophany, an irruption of the sacred that results in detaching a territory from the surrounding cosmic milieu and making it quantitatively different". The distinction between the profane and the sacred may contain the following elements: reverence; deference; sanctions, prohibitions and rules of conduct; demands for offerings; territorial demarcation that serve as centres of pilgrimage [[3]:15,52; [2]:29–36; [4]:368; [5]:15; [6]:11,13; [7]:1]. These means were evolved to keep the profane apart from the sacred [[3]: passim; [6]:11; [8]:20–22]. One should bear in mind that the sacred cannot exist without the profane, for the former needs to be constructed and protected from the latter [[9]:50]. Trees are very common as sacred objects as and as leading landmarks of sacred places [[10]: passim; [11]: passim; [12]: passim; [13]: passim; [14]: passim; [15]: passim].

In the Muslim world, as well as in the Middle East, sacred places are closely related to the veneration of saints [[16]: passim; [17]: passim; [18]:passim; [19]:passim; [20]: passim] and, in many instances, sacred trees are connected with sacred graves/shrines and share the same supernatural powers, to grant divine blessings, to cure and to punish the offenders against the saint to whom the tree is dedicated and who endows them with their miraculous powers [[19]: passim; [15] passim; [21]:passim; [22]passim]. So it is not surprising that many of the customs and ceremonies which are performed, in general, in sacred places, are performed also at the sites of sacred trees. Frese and Gray [[23]:32] have already stated,"Sacred trees have a ritual significance. The trees and their meanings may be incorporated into rituals of curing, initiation, marriage and death. Trees used in any of these contexts stand for the divine and represent the sacred beliefs being honored through the ritual". Eliade [(1963,[4]:268] has already stated : "No tree was ever adored for itself only, but always for what was revealed through it, for what it implied and signified". Sacred trees are, thus, treated as any other sacred places and one may expect to see common customs related to sacred trees as well as to any other sacred places.

This work studies the present-day rituals and customs related to sacred trees in Israel; it is based on personal interviews as well as a field survey. Some of the customs related to sacred trees have already been considered elsewhere (seeking for asylum, taking oath, deposition of properties [[22]: passim]; tying rags [[21]: passim] and the hammering of nails [[24]:7]. Although the division is somewhat artificial, the present paper is more concerned with social as well as religious issues. It also includes some very brief additions to the former papers resulting from our continuous field study since 2000.

## Methodology

The field study (1999–2006) centred on thirty-one Arab, Bedouin, and Druze villages in the Galilee. Informants were asked about the customs and ceremonies performed near or under sacred trees. The survey covered 98 informants, consisting of 34 Druze and 64 Muslims (45 Arabs and 29 Bedouin). The distinction between "Arabs" and "Bedouin" has been made in an attempt to examine whether there were any different traditions regarding sacred trees which may reflect the different origin of nomads versus settled village people. We took "Arabs" to be people settled in their villages for several centuries, and "Bedouin" as people who originated in the deserts of Israel and Jordan, and who migrated to the Galilee in the last three centuries, and were nomads until the end of the 20^th ^century [[25]:30].

The Druze are an eastern Mediterranean religious group first established in Egypt in the 11^th ^century [[26]:3]. Today they are concentrated in Lebanon, Syria, and Israel [[26]:8–14]; their belief in the revelation of God in the form of a human being is considered the most important fundamental principle of the Druze faith [[26]:15] which is not a ritual and ceremonial belief in essence, but rather a neo-platonic philosophy [[26]:17].

The survey excluded Christians, who hardly believed in sacred trees while, in the Jewish sector, the adoration/worshipping of trees is a new trend of the last two decades and almost all the worshipperd trees are already known as old Muslim sacred ones in the vicinity of graves of supposed historical righteous Jewish personalities.

In each village we carried out a preliminary survey to locate the more knowledgeable people in advance, and we also chose important religious leaders to examine their attitudes to the veneration of sacred trees. The informants were chosen mainly according to their knowledge of common/local traditions and/or religious status. The average age of the informants was 57.7 (+/- 14.8) years. Respondents were 86 males and 22 females (in general women are reluctant to be interviewed and, when they agreed the interview was held in the presence of other family members). Because of the refusal of most of the informants to be videotaped or recorded, the study is based entirely on oral interviews and field notes taken on the spot. The interviewees were asked what are the customs and manners performed at the sacred tree. We also surveyed 26 sacred trees near which active worship takes place today and could be observed. (Numbers printed in bold in the table and the text indicates how many informants related to a specific issue).

## Results

The results concerning religious and community issues are presented in Table 1 family/personal issues and respect of the tree in Table 2.

**Table 1:** Religious/Community Issues

**Issue**	**Druze** **(n = 34)**	**Bedouin** **(n = 29)**	**Arab** **(n = 45)**	**Field observ ations** **(n = 26)**	**References from the Middle East & adjacent areas**	**References from other Countries**
Religious/social/leaders & meetings	**23.5**	**31.0**	**26.6**	**0**		Russia (31):128); Sierra Leone (138):311); Tanzania (34:16, 35:41); India (139:238, 140:17); Korea (141:9); Indonesia (142:45); American indians (143:13)

Animal sacrifice (when vows come through)	**50**	**48.2**	**42.2**	**7.6**	Lebanon (144:334); Syria (145:290)	India (40:344)

Pilgrimage/Zayara/family gatherings	**11**	**13.8**	**13.3**	**23.0**	Israel (146:113)	

Rain-making ceremonies	**0**	**27.5**	**15.5**	**0**	Arabia (62:11–12); Algeria (147:41); Tunisia (54:521,519; 147:242) :	Ancient Rome (12: I:232); Tanzania (34:16,37,41); Kenya (148:143,145; 47:139–140); Sudan (29:464); Uganda (149:38; 48: passim); East Africa (150:263); Tanzania (52:476–477); Zimbabwe, (125:6; 151:379; 46:362; 358, 361; 152:291); Ruanda (153:82,86); Chad (57:229–235); Senegal (35:420); Mozambique (122:237); Centra Africa (49:53–55) India (40:330; 51:67; Papua (53:386,387)

Passing judgment under the tree	**0**	**27.5**	**4.4**	**0**	Ancient Israelites (Judges, 4:5)	Germany (154:85); Morocco, France, Switzerland (14:145); Somalia (155:169); India (27:59); Japan (156:28)

Sulkha (conciliation of quarrels) under the tree	**0**	**20.6**	**0**	**0**		

Leaving water under the tree	**0**	**13.8**	**8.9**	**23.0**	Kurdistan (157:384)	Scotland (99:75,76); Russia (106:322); India (115:20, personal observation. 10.2.07);

Decoration of tree (leader's pictures)	**14.7**	**0**	**0**	**7.6**		

Official religious ceremconies under the tree and/or grove	**0**	**0**	**0**	**0**		Ancient Greece (158:219 note 417); Guinea – Bissau (159:389); Siera Leone (139:311); Zimbabwe (61:6); Liberia (160:1222); Mozambique, (122:9); Inner Mongolia (129:283); Okinawa (161:48,49)

Using parts of trees tree's in daily religious rituals	**0**	**0**	**0**			India (115:20, 93: passim)

Sacrifice to appease supernatural beings					Biblical times Hosea (4:13)	Ancient Crete (162:128); Ancient Greece (163:74; 158:220); Russia (31:94; 106:321, 164:114); Siberia (165:853); Inner Mongolia (129:281); Ghana (166:69); Liberia (160:1222); Nigeria (167:425); East Africa (168:52, 148:263); Kenya (169:155); Liberia (160:1222); India (170:294);

Performing rites of passage	**0**	**0**	**0**	**0**		East Africa (45:4; 171:57); Kenya (169: passim); Liberia (160:1222); Sierra Leone (172: 159,160); West Africa: (173:63); India (76:91, 174:50; Nigeria (175:80,81);

**Table 2:** Family/Personal Issues/Respect of the tree

**Issue**	**Druze** **(n = 34)**	**Bedouin** **(n = 29)**	**Arab** **(n = 45)**	**Field obser-vations** **(n = 26)**	**References from the Middle East and adjacent regions**	**World references**
Rag tying	**73.5**	**68.9**	**84.4**	**100**	Middle East (21 and references therein).	Many countries all over the globe (See 21, and references therein)

Placing object for the absorbing of Barakeh/divine blessing	**38.2**	**37.9**	**71.1**	**57.6**	Israel(22: Passim, 176:48,51)	

Personal vows/requests/petitions for health/wishing tree/	**58.8**	**62.0**	**71.1**	**15.3**	Israel (19: passim,, 20, passim, 21:passim, 22: passim, 177:176, 136:79); Syria (145:296, 178:173)	Scotland (179:499); Estonia (180:4) ; Serbia (30:97); Russia (106:322); East Africa (45:4, 150:263); Sierra Leone (126:48); Guinea – Bissau (159:389); Chad (181:248); India (182:353, 183:55–59, 184:43); Japan (185:23; 186:10); Australia, New Zealand (14:164)

Weddings	**17.1**	**51.7**	**28.8**	**15.3**	Israel (20: passim)	India (76:91, 39:175, 187:62)

Lighting candle/lamps	**23.5**	**24.1**	**31.1**	**65.3**	Israel (136:79, 176:43); Lebanon (144:34); Turkey (88:216, 89:80, 188:41)	Balkan (188:41); Russia (31:94); Uganda (189:461): Armenia (190:II:173); India (93:87)

Hammering of nails	**8.8**	**17.2**	**8.9**	**23.0**	Israel (:24:7–8); Egypt (95:27); Turkey (89:80); Kurdistan (88:216)	Europe (84: passim); Russia (106:320); Ireland (105:195). India (92:97, 93:182); West Himalaya (94:xxi)

Placing stones on/under the tree Leaving stones on the tree or in heaps/cairns	**2.9**	**10.3**	**8.9**	**26.9**	Ancient Israel (101:212); Israel (177:76); Muslim world (88:211)	Russia (191:114); India (103:19, 1921:457, 458); Korea (60:44);

Incense (as a part of a personal form of praying)	**5.9**	**17.2**	**24.41**	**19.2**	Israel (193:359); Syria (144:297)	India (51:66); Armenia (190:II:173);

Funeral/burial under the tree/at the sacred grove (also of leaders and heroes)	**0**	**24.1**	**20.0**	**23.0 **Existing tombs/cemet eries	Ancient Israelites, (Genesis 35:8); Israel (137:37, 136:74).	Mongolia (129:280); Zimbabwe (194:200, 195:164); Kenya (196:6); Ghana (197:34, 198:225); Mozambique(122:229); Tanzania (199:59); Okinawa (161:48);

Tree Embracing/touching/kissing	**55.9**	**6.9**	**4.4**	**7.7**		Ancient Rome (200, XVI: 242);

Leaving objects/food under tree as charity	**26.4**	**20.6**	**15.5**	**30.7**		

Praying while passing near the tree	**0**	**6.9**	**7.7**	**0**		

Cleaning around the tree	**5.9**	**10.3**	**2.2**	**23.0**		

### Wedding preparations under sacred trees

In some villages there are sacred trees which are called "Sajarat el Orsan (the groom's tree, **8**) or "Sagarat el Arus (The bride's tree, **7**). These names reflect the old custom of performing weddings under these trees. Just before the ceremony at the groom's house he was brought to the sacred trees for final preparations (Zaffa). Mats were spread under the tree and food and sweets were offered to the guests. A group of males surrounded the groom and washed him, he was then dressed in beautiful clothes and his friends used to encourage him while the women gathered nearby singing special songs. Sometimes horse races were held on the same occasion. Later the groom was brought in a special ceremonial parade to his home in which the official meal was held. (This ceremony or parts thereof were common in many villages, **12**, all of which are Muslim). Weddings under sacred trees are known also among the Druze; here it is more of a kind of feast or celebration – but without special attention being paid to the groom, **10**). When people were asked why the ceremony was held under the tree some (**7**, Arabs) they said that it was to get a blessing, while others (**5**, Bedouins) mentioned that the large solitary tree was a good place for gathering under as it offers much shade in the summer and is a good place for horse racing. The Druze (**5**) mentioned the pleasant and spacious area around the tree as the reason for choosing it as the place for the celebration, while others (**4**) indicate that it was done "for a blessing".

### Rainmaking rituals

The rainmaking ceremony at the village of Kaukab Abu el Heija, in the Western Galilee, was so famous that people from other villages in the region used to take part and each delegation brought its special flags which were assigned for this specific purpose. When there was a rain arrest, the rainmaking parade was leaving the village from the sacred saint's shrine of Sheikh Sa'eed (in which the flags and the musical instruments are deposited even today) to the close mountain (Mt. Atzmon). The participants were equipped with their flags and special musical instruments. On the way they stopped at a sacred tree (Christ Thorn Jujube, *Ziziphus spina christi*, the tree had already disappeared) and they put the "rainmaking flags" near this tree. Here they read the opening chapter of the Quran (Surrat el Fatikha) while asking another saint (Abu El Heija, the local saint, buried on an adjacent hill) for a permission to continue the ceremony. Then they approached his grave and circumambulated it seven times while praying.

The parade continued to another sacred tree; underneath it was the grave of Sheikh Ottoman (the tree of *Pitacia lentiscus *and the grave has already gone) and circumambulated it seven times with the flags. Then they climbed the mountain which is close and prayed near an impressive tree, an evergreen oak (*Quercus calliprinos*), circumambulated the mountain peak and prayed for rain. They then returned to the village in a special track (along a special path) which is called "the way of the musical instruments and the flags" and the rain begun soon. The last ceremony was held around 1953 (**8**).

In other villages we heard that rainmaking ceremonies and praying were carried out near sacred trees, they included special songs and prayers (which may have varied from village to village) and sometimes included the sprinkling of water. (**10**, from six different villages, all of which are Muslim), these ceremonies came to an end over the last three to five decades.

Rain making ceremonies at the Druze sector are quite rare and performed (mainly as water pouring and special songs, **8**) by woman and children in the streets and are never related to sacred trees. The religious leader considering rainmaking ceremonies as a kind of an intervention in God's action. A popular saying, reflecting this view, says "My son doesn't look at the cloud, God's mercy is closer" (Salekh Khatib, 22.4.07. Pers. comm.,)

## Discussion

### The tree as a social centre

In many cases it is easy to attribute meetings under the tree not to its sancticity but simply because many of them are very large and give a lot of shade (**5**). As a rule, in Israel, there are no official religious meetings under a tree and it is not a centre of communal worship as in many polytheistic religions.

### Judging under the tree

Judging under trees is known from Biblical times (Judges 3:5). Hamilton [[27]:59] reports that, even today, no Hindu or Buddhist shrine is completed without a sacred tree planted nearby. These large trees (pipal and banyan) have become natural assembly points for village meetings, community events, and the dispensing of justice. In central Europe, the most venerable oak in many towns and villages became a site of justice where the magistrate sat when he passed judgment [[14]:145–146], and those trees were preserved as "justice trees" [[27]: 61–62].

### Sacrifices under the tree

Sacrifices have been carried out since time immemorial in almost in every known culture, to propitiate gods and spirits, to restore the divine connection and to wipe out the offence, [[28]: passim], according to this author [[28]: VII] "...The world was once seen to be alive with gods and spirits needing nurture and propitiation. If offered the right sacrifices, they would dispense aid and grant special favours". Because sacred trees/groves were regarded as the abode of spirits/gods/supernatural beings (see review in[15]) it is not surprising that sacrifices were offered under these trees and groves in various religious ceremonies [[12]:1926:passim; Sudan, [29]: 458,461; Slavs in Europe, [30] :97; Russia, [31]: 66,113; Nigeria, [32]: 42,46; Ghana, [33]:599,603; Tanzania, [34]:16; Senegal, [35]:420; Senegal, [36]:5] and even human sacrifice in the past (Nigeria, [37]:74; India, [38]: passim; [39]:29,245,484;565,568; [40]:344; Thailand, [41]:107; Indonesia, [[42]:165].

In the world of Islam "the great majority of sacrifices are made in payment of vows furnished to some saint. As soon as the animal is killed, it ceased to belong to the one who offered it and becomes the property of the Weli (the local saint)" [[43]:172]. The common tradition is to share the meat with other people including needy ones [Palestine, [19]:165; [20]: passim, Morocco, [44]:457]. Such a sacrifice is made at saints' graves and shrines [[19]:154–174; [20]: passim; Morocco, [44]:457] as well as under sacred trees (Table 2). At present, a goat or sheep maybe is dedicated to the saint as a part of the fulfillment of a vow and as a form of thanks for the granting of a personal request [[19]:158, the animal is slaughtered at the saint's shrine [19]:160; [20]: passim]. near the sacred tree and the meat is given to the needy and/or used by the family (**48**). This kind of sacrifice is also known from: Nigeria [[37]:52; East Africa, [45]:4; Morocco, [44]:457 and Palestine [19]:158]. This custom has nothing to do with a sacrifice to any supernatural being that is related to the sacred tree or the grove in which sacrifices are part of the official religion (see above).

### Sulkha

Sulkhas being conciliations between families, especially when serious quarrels or murder were involved. In the village of Arab a' Shibli (in the foothills of Mt Tabor) there is a special tree (*Quercus ithabusresis*) named Al Mizar (= the visits) under which the local judges used to sit regularly until around 1950 (**17 **informants from this village). Near this village there was another oak tree (named "Sajarat el Bahta") near which Sulkhas were carried out (**6**). The act of a "Sulkha" under the tree seemed to prevail, in the past, more in Bedouin than in Arab villages and was not found by us among the Druze.

So far this custom (performed under a sacred tree) was found by us only among the Bedouin. Sulkha procedures are very common among the Arabs and are held generally at the leader's (Sheikh's) house. It seems that among the Bedouin it is carried out under the tree especially because it is a well-shaded place; not one of our informants has pointed out a special connection between the sacred tree and the saint that is related to it.

### Rain making ceremonies

Rainmaking ceremonies are known worldwide in many diverse communities [[12]: passim]. A rainmaking ceremony may contain one or more of the following elements: water pouring, imitation of rainfall and thunder prayers, use of musical instruments/special songs, sacrifices and the use of special objects for these ceremonies. The ceremony is carried out by special members of the community; it is a secret ceremony, which takes place in special places frequently sacred ones, there is the performing of a special procession, as well as special costumes. These elements or parts thereof were reported from many countries [Palestine, [19]: 219–234; Zimbabwe, [46]:356; Kenya, [47]:139–140; [45]:4; Uganda, [48]:62,71,72; Central Africa, [49]:53–55; Sudan, [50]:54; India, [51]:67; [34]:330–332; [52]:476–477; Papua, [53]:390; Tunisia, [54]:passim; India, [55]:143–174; Japan, [56]:1–30; Ghana, [33]: passim; Chad, [57]:230]

One may suggest several reasons why rainmaking ceremonies are performed near/under sacred trees/groves/forests and/or large trees:

1. The tree/grove is associated with the rain/thunder god. According to Ruppert [[55]:143] "the sources of rain were conceived in various ways, most commonly as one form or another of deity, especially associated with the heavens or with creation in general". The tree is the abode of the sky/cloud/rain/thunder divinity who is "in charge" of rain [Ancient Greece, [58]:8.38.4; Pagan Europe, [59]: passim; [60]:31, 34–36; Uganda, [48]:59, Papua, [53]:388; Sudan, [50]:53; Central Africa, [49]:53–55; India, [51]:67; Zimbabwe, [46]:358,361]. Thus, it is not surprising that rainmaking ceremonies were carried out under the sacred trees (Table 1) as they are considered to be the abode of the supernatural beings which bring the rain.

2. Gerden & Mtallo [[34]:45] explain the connection between sacred trees and rain making ceremonies "The trees... are important for the formation of clouds that move up to the peak of the mountain... According to local belief, when clouds ascend it is a sign that rains will start. If the trees are cut, no clouds would come up to the mountain top and there would be no rains"

3. The tree is the abode of the supernatural beings that protect the village and take care of its prosperity. Chidahakwa [[61]:6; Zimbabwe] stated:" Some sacred trees are places were rain ceremonies are conducted and are regarded as the protectors of the village and the seat of the ancestors" [also in Chad [57]; 235].

While considering the possible connection between sacred trees and the rainmaking ceremonies in Israel, one should bear in mind that these ceremonies were common, in the past, in almost in every village, regardless of any trees [[19]:219–233]. In general, throughout the Moslem world, rainmaking ceremonies are performed at saint's shrines [[18]: II: 256]. Because sacred trees are regarded as the abode of the saint's soul [[19]: 151; [15]; passim], rainmaking ceremonies could be regarded as another aspect of using the sacred tree as a channel to the merciful god asking for his favour as it is done in many other cases of trouble [[19]:151 [21,15,22]; passim].

Palmgrave [[62]: I: 11–12] described a rainmaking ceremony in Arabia around an *Acacia *tree which also includes prayers and dances and mentioned that it was a pre-Muslim relic. Thus it is another manifestation in which tree worship of old local deities were replaced, in the Muslim world by a veneration of the saint. [[16]:316; [19]:151; [15,21]: passim]. According to Westermarck [[17]:122] Muslim Saints "may influence the power to produce rain as one of the gifts most frequently ascribed to them" [see also [19]:271 who mentioned that saints may prevent rain]. Canaan [[19]:219–230] mentioned that requests for rain are made frequently at saint's shrines, some of whom are known to be more efficacious than others in giving blessings and their shrines are preferred for rain processions [[19]: 227]. This also explains why these ceremonies were confined to Muslims: the Druze do not consider sacred trees as an abode of any soul [22]. Sacred trees are not mentioned in relation to requests for rain in the entire chapter that is devoted to rain procession in Canaan [[19]: 219–234].

Rainmaking ceremonies near sacred trees are the only situation that the present author is aware of, in which a request using sacred trees for asking of a favour is for the whole community and not only for the welfare/health/benefit of the individual person and/or his family. Public petitions for the whole community (which are not related specifically to rain-making) are held in many polytheistic religions under sacred trees or groves which are the abode of the protecting god of the village [[63]:113; Kenya, [64]:89; Mozambique, [65]:14; Laos, [66]:324; China, [67]:352; [68]:6; [69]:131–132; India, [70]:8; [71];345; [72]:66–68; [73]:2001; [74]:151:315–319, [75]:384, [76]:96; East Africa, [77]:414,432; Ivory Coast, [78]:370; Nigeria, [79]:290,292,293; Ghana, [80]:366; [81]:159; Timor, [82]:90–99; Vietnam, [83]:113; India, [72]:67 and Chad [57]:230] one of the possible punishments for the violation of such trees is rain stopping.

### Rag tying

It seems that the custom of tying rags onto sacred trees exists in almost every known human culture, going beyond the borders of religion, geography and time [[84]:passim; [85]; I: 111; [12]:7–96, see [21] for a review]. Rag tying is largely distributed in the Moslem world [[16]:316]. Rix [[86]:32] noted that clothes that are left on sacred trees are not just gifts in the ordinary sense; rather, they are channels connecting the worshipper with the object or person worshipped. In the Moslem world, rags, used clothes, yarn and threads are tied, in the shrines or tombs of holy figures (Wellis) and on objects around them such as sacred trees, the wire netting which covers the windows of saints' tombs, fences, [[86]:180].

Curtiss [[43]:92; [18]: 562; [19]:103] in order to get the saint's divine blessing ("Barakeh"). Rix [86]:32] mentioned, "Holiness is, indeed, to the Palestine peasant a sort of liquid which may be absorbed by physical contact. The man who hangs a rag upon a tree will take from it and wear about his person another rag which has become soaked with the virtue of the place by hanging there..."

Dafni [21] found seventeen reasons for tying rags on sacred trees worldwide, twelve of which were recorded in Israel: Five reasons (the breaking of an existing oath, to mark a blessed tree, to mark the road to a blessed tree, to ask for permission to pick fruit and to leave rags for needy people) which are endemic to the Druze. Two reasons (to pacify the tree's spirit and a charm for new clothes) were previously reported from Israel but were not confirmed. Three other reasons (transference of one's illness to the tree, using a rag as a visiting card and to pacify the tree's spirits) are also known beyond the Middle East. Other reasons (such as ensuring good crops, offerings to the tree's deities/spirits, pacifying the ancestor's spirits, commemorating a death, pacifying a tree's spirit while picking fruit) were never reported from the Middle East and are connected with polytheistic religions.

### Hammering nails into sacred trees

Hammering nails as well as hanging clothes are "tying" rituals, whereby the person seeks healing or a solution to problems by transferring his or her illness or problems to the tree, or to whatever object the clothes are hung on or nails hammered into. Such "tying" is one of the best known and commonest beliefs practised throughout the world among Christians, as well as among Muslims and their predecessors in the Middle East [[88]:213; [89]:262; [21]]. Hartland [[84]:459–460] has already identified the common background of hanging rags and putting nails on sacred trees as "generally the attainment of some wish, or granting of some prayer, as for the husband, or for recovery from sickness".

In several countries nails are hammered to a sacred tree to transfer the pain or illness into the tree [England and Germany [90]:493; Kurdistan, [88]:216; Europe, [84]:58; and Turkey [89]: 176,262; [91]:128].

In India the emetic nut tree (*Strychnos nuxvomica*) is considered the prison of all demons. Occasionally such trees can be seen with trunks full of nails as a precaution against demons. If a demon or bad spirit dares to attack a human, the exorcist forces it back into the tree with a nail. With each nail driven in the demon declares that it will not attack again. Nailing the demon into the tree trunk is the best way to give it a life sentence [[92]:97; [93]:183]. Sacred trees in the West Himalayan region are the object of a similar custom: travelers hammer nails into the trunk when passing by as a protective step against diseases, death, and any damage to their sheep, cattle, or crops. The explanation for this act, according to traditional belief, is that it dispels evil powers [[94]: xxi].

In Egypt, nails driven into tree trunks signify the prayers of the believers. People come to sheikhs' trees to be cured of headaches or other ailments. In asking the sheikh for help, they hammer nails into the trunk and wind some of their hair around the nails [[95]:56]. A ceremony of this kind was recorded at sacred graves in Turkey [[89]:80]. Some are of the opinion that this was a gypsy tradition introduced from India [[96]:147]. Our informants (**8**) mentioned that nail hammering is done against the evil eye as "A nail in the eye of Satan" (**4**).

A square in central Vienna is named *Stock am Eisen*, which means literally "iron on the stick". A glass case stands on one of the corners of the square containing a replica of a piece of wood into which some nails have been driven. A known tradition from the 16^th ^century relates that any apprentice who completed his duties in the town would hammer a nail into a tree that grew in the square for good luck [[97]:21; [98]:99].

Some authors mentioned that nail hammering is done just to fix clothes/rags [Europe, [84]:453,454; Scotland; [99]:75; Yemen, [87]:213–214] or money to the tree [[98]: 75].

### Leaving stones on/under the tree

Stones are put in certain places when people died as a token of honour to the deceased [Ireland, [100]:43; Morocco, [18]: II: 549; [101]:212, Israel (Bedouins: Negev) [102]:76], this custom is very common today in Europe as well as Israel (personal observation].

In the Muslim world it is common to put a stone on or under sacred tree "when a woman yearns for a child, when a peasant longs for rain, or when he yearns for the restoration to health or his horse or camel" [[88]:211]. According to Westermarck [[18]:1: 75–76] cairns are placed under sacred trees for curing just as they put rags or threads. Canaan [[19]: 75] mentioned that stones are placed in heaps at saints' shrines to show piety and as a visiting card. In India believers put stones under a sacred tree as part of a worship of a local deity that dwells in the tree [[103]:19] and in Korea as an offering [[60]:44]. Not one of our informants was able to explain why stones are left on or near sacred trees although stones on sacred trees are quite common (Table 2).

### Leaving money on/under the tree

In Israel, people used to leave money under the tree as well as in saints' graves (**5 **informants, Leaving money in graves is a very common custom in the Muslim world [[18]: II: 502; [104]:159] as charity for the needy (**6**). Money is left on trees when a wish is made as an offering to the supernatural being to ensure the fulfillment of the personal request and for wishes and good luck [Scotland, [98]:75,76; Ireland, [105]:195; Europe in general [84]:passim; [106]:322].

### Weddings

People used to arrange weddings under the sacred tree to receive blessings from the saint to whom the tree is dedicated (**24**) and, also, just because it was almost the only large available tree that gave considerable shade (**5**, all of which are Bedouin who stressed that the tree was a meeting point because of this very reason). In some villages there are special sacred which were used for the pre-wedding ceremonies. Weddings under a sacred tree can still be seen in the village of M'ghar (Lower Galilee) under the huge *Ziziphus spina christi *of Sheikh Rabis. This custom was more common among the Bedouin in rural areas but quite rare among the Druze whose religious leaders are strictly against this custom (**4)**.

### Taking vows

Vows are taken under a sacred tree just as they are in saints' shrines [[19]:132–133]. This is frequently manifested by tying rags in the shrines or on sacred trees [see [21] and references therein]. The religious belief is that these places are regarded as channels between God and his believers through the saint's mediation in his place [shrine or a tree, see [22], passim; [15]: passim; [19]:35–38; [16]: 21].

### Incense

According to Groom [[107]:1] "Incense has had a continuous religious significance throughout the entire expanse of history from the first civilization to the present day...It was used ...to purify and to please gods and as an offering to the gods". The use of incense was very important in Jewish traditions in Biblical times [Bible citations; [108]: passim; [109]:71]. Burning incense under a sacred tree is already mentioned by the prophet Hosea [[4]; 13] when he complained of the practising of tree worship in Israel.

Groom [[107]:2] explains the use of incense "The spreading of the smoke and fragrance of incense and the visible movement of the smoke upwards towards the heaven has given it a symbolic relationship to prayer, making the offering synonymous with worship". When our informants were asked why they used incense (under the sacred tree) the answers were: for barakeh (**16**), to honour the saint (**9**), against the evil eye (**4**, as it is known in Jewish communities [[108]:13] as well as in the Arab traditions [[19]:148] and for a good odour (**5**). The common incense stuff is dried leaves of *Salvia fruticosa *which is frequently used locally also in rites of passage [[110]: passim]. The burning of incense is very common at saints' shrines [[107]:2–3; Palestine [19]:148,249; Morocco, [18]: I: 123].

### Candles/oil lamps lighting

Candles, as well as other kind of light sources, are used all over the world in religious ceremonies as explained by Weightham [[111]:59]. "The presence of light as the manifestation of the holy spans multiple religions. Light, through its presence or absence, sets apart the sacred from the profane"

Candles and oil lamps are lit on the graves of righteous people and saints by Jews as well as Muslims in Palestine [(Moshe Yerushalmy 1769 in [112]:431; [113]:38] as well as in Morocco [[44]:459]. Candles and oil lamps are also lit at saints' shrines [[19]:96]. Candles are lit in general while making a vow [[19]:132; **23**]. personal praying (**18**) or as a regular commitment (generally on certain days of the week, especially Thursday night) to the saint as a result of a vow (**7**)

Westermarck [[18]: I: 302] explained the use of candles" The Jinun (devils) are fond of darkness and terrified by light. The burning of candles is therefore a means of keeping them away". Our informants explain candle lighting as for Barakeh (**7**) and as thanks (**6**) to the tree/saint as it is also done in a saint's shrine [Canaan, 1927–8:19:132].

### Use of sacred parts of trees

In the Middle East, parts of the trees may be taken as talismans/charms/amulets/medicine because the tree had the divine blessing of the saint " ("Barakeh") to whom the tree is dedicated [[19]: passim; [20]: passim; see [22] and references therein]. The influence is based on magic contact; this has also been noted in Europe [[114]:18]. moreover, religious objects made from the wood of the sacred tree are known in other parts of the world, especially in India [[115]:44].

Sacred trees are believed to have magical curative powers in pagan Europe [[116]:276–277; [117]:42–45; [118]: I: 169–193]. In Israel, even species of some plants or parts of it that are not known as having medicinal properties, such as the leaves of a sacred oak (*Quercus calliprinos*), are regarded as omnipotent forms of medication when administrated externally as a poultice (**6**) or as a decoction (**8**). Leaves of a "secular" oak are not used for healing. Clearly, the leaves acquire the healing powers only when granted by saints; just as actual medicinal plants gathered in the vicinity of the sacred tree are more potent than their conspecifics (**5**). Most of the uses of sacred trees for divine blessings or cures or as talismans (Table 2) are based on a magical contact.

In our survey it was found (Table 2) that individual people may use parts, especially the leaves of the tree only as carriers of the saint's divine blessing (Barakeh). Sacred trees are never used as a part of an official religious ceremony.

The use of parts of sacred trees (especially specific species) is very common in India, especially when the specific trees are sacred to a certain deity [115]: passim; [11]: passim; [93]: passim] as part of the fulfilling of daily rituals (especially leaves and flowers), also in Nigeria [[119]:125,129].

### Charity

Visitors to sanctuaries, in Palestine, used to leave objects in honour of the saint as votive objects, as part of a fulfillment of a vow or for use by later visitors; these included candles, oil for lamps [19]:144–146, also personal observations], incense [19]:148] and matches [19]145]. It is unique to the Druze believers to leave used clothes at sacred trees as a charitable gift for use by other people [21].

### Decorating the tree

The Druze sometimes put pictures of their religious leaders on sacred trees (Table 2) as they used to do in their house of prayer (Hilwe) and other sacred places (personal observation.). When they declared a "new" tree as sacred on Mount Carmel [see [15]] they decorated it with such kinds of pictures. The reason given was "hanging pictures brings blessings" (**4**). So far this custom has been found only in the Druze sector.

In addition to the great fear of punishment due to harming or making sacrilegious utterances about the trees [see [22]], there are many gestures which show the deep respect for the trees; these are performed while approaching or visiting the tree such as a ban on defecating or urinating near the tree (**3**), swearing (**3**), cleaning around the tree (Table 2); a need for personal purifying (e.g. washing before visiting the tree as is done before a visit of the mosque) (**5**); the saying of a special chapter from the Quran (**18**); and it is forbidder to leave ant dirt under the tree (**5**).

### Kissing and embracing of the tree

This custom seems to be almost exclusive to the Druze. They (13) explain that kissing the "blessed tree" [see [15]] is to receive a blessing as the kissing is done in a sacred grave **6)**. The same is regarded for putting the palm of one's hand on the tree (**5**). In India, people embrace a sacred tree in order "to get their desires fulfilled" [[103]:23].

### Sacred trees, saint's graves

In the discussion about the reason/s for the sanctification of trees [[15] passim; [18]: passim]. it is agreed that there is a similarity between sacred trees and graves of Muslim saints (Wellis). The spirit of the Welli dwells in his grave or his shrine or in a tree which is dedicated to him. It is not surprising to see the close similarity between the ceremonies and rituals that are performed in a saint's shrine/grave and at a sacred tree.

Many of the rituals and ceremonies that are held at the sacred tree first take place at the saint's shrine or his grave. Analysis of the places where ceremonies and rituals are performed [[20]: passim; [19]: passim] shows that the sacred tree is only regarded as such as a kind of a "default"; there is also a kind of hierarchy among the saints: some of them are regarded as more powerful in their supposed powers. For example Canaan [[19]:133] has already mentioned "Vows are not only made to sanctuaries where a *maqam *(saint's shrine) and a tomb are found, but every other shrine......Naturally what is vowed to these shrines – stones, trees, caves, springs, etc. – is as a rule much inferior to what is offered to the anbiya (= prophets). Offerings to supposedly holy stones, trees, waters, etc., are another connecting link with primitive religions". When it possible sanctuaries and saint's shrines are preferred to trees [[19]:passim; [19]: passim]. Thus it is not surprising that sacred trees replaced graves and shrines in their absence or when they were more available at a local level.

### Monotheistic vs. polytheistic sacred trees

In many polytheistic communities there is a close relationship between sacred trees/groves and burial places which show the close links between ancestor's souls and tree worship [Taiwan, [120]:5,III,I; China, [121]:133; Mozambique, [122]:131; Ghana, [123]:41; [80]:366; Madagascar, [124]:19–20; Zimbabwe, [61]: 6]; Sierra Leone, [[125]:47]; Rhodesia, [126];102; Kenya, [127]:1350; French Guinea, [128]:14; Inner Mongolia, [129]:277,280; India, [130]:242; [131]:332; Laos, [132]:4; Indonesia, [[133];310,318; Papua, [134]:72]; Australia, [[135]:163].

Although there is a connection, in monotheistic religions, between sacred trees and burial sites it is not related, nowadays, to any form of ancestor worship. In Israel, sacred trees are frequently located in cemeteries, sometimes heroes or important people are buried under the sacred tree as a token of special homage [[136]:74; [137]: passim]. This custom is not related to ancestor worship (see references above) and sacred groves as the community burial places [see references above and in [15] and references therein] are unknown.

Dafni [22] has already noticed that in the Middle East, as well as in Europe, tree worship today is practised by individuals making personal petitions. Tree worship is, by no means, a part of the official monotheistic governing religion. In Israel there are no performances of regular religious ceremonies, as they may be held in a *maqam *(a saint's shrine) [[19]:98] or of, course, in a mosque that is performed at a sacred tree. Nevertheless, in many polytheistic religions, tree worship is a part of the official worship and is performed at a community-based level [see discussion and references in [22]].

### The uniqueness of some Druze customs

Some customs such as rainmaking ceremonies, burials, pronouncing judgment, conducting a Sulkha, and leaving water under the sacred tree are absent in the Druze sector.

As far as the author is aware, rain-making ceremonies are almost unknown in this sector in relation to sacred trees, although they were previously common in the villages (**6**).

The Druze believe in the transmigration of souls: a person's body is a kind of clothing for the soul and, with death, the soul passes to the body of a newborn child [[26]:60]. The Druze never consider sacred trees as an abode for the souls of righteous figures and certainly do not relate trees to graves [see [22]]: for further discussion. However, some Druze ascribe supernatural powers to sacred trees [22]. Their fear and admiration of such trees are of the same magnitude as in the Muslim sectors [(Tables 1 and 2, and [22]]. While the Muslims credit the miraculous powers (e.g., the trees' immunity to fire) to the souls of Wellis or of God, the Druze ascribe these powers to their prophets or religious leaders themselves [22].

## Conclusion

When comparing the customs and ceremonies which are held, in many cultures, in sacred places and religious shrines as well as under sacred trees and groves one may come to several conclusions:

• 1. Sacred trees are just another kind of sacred place with all their metaphysical as well as physical manifestations. In our region, sacred tress are frequently related (by Muslims) to the shrines of saints

• 2. There is not even one ceremony or custom that is peculiar only to a sacred tree and is not performed in relation to other sacred places (such as a saint's grave or mosque).

• 3. Few customs (e.g. the settling of quarrels = Sulkha), leaving objects to absorb the divine blessing and leaving objects for charity) seems to be characteristic of the Middle East

• 4. Sacred trees were never recorded in modern times, in our region, as centres for official religious ceremonies including sacrifices, or places for the performing of rites of passage.

• 5. There are some variations among the different ethnic groups: The kissing and worship of trees is more common among the Druze while burial under the tree, leaving water and rain-making ceremonies under the tree was not recorded in this group. Passing judgment under the tree is more typical of Bedouin communities in which the sacred trees were commonly used as public social centres.

Most of the customs/manners related to sacred trees and groves are ubiquitous and can be found throughout human history. Some of these, such as family gatherings, conducting a Sulkha, the leaving of food, the leaving of objects to absorb divine blessings and leaving objects for charity) seem to be characteristics of this region, while the performing of official religious ceremonies under the sacred trees was never recorded in present-day Israel and is typical of the old Semitic religions [[43]: passim]. Today, these ceremonies exist mainly in polytheistic religions.
